# Cumulative Incidence Rate Score for Alzheimer’s Disease Risk is Associated with White Matter Hyperintensity Volume in Older Adults Without Dementia

**DOI:** 10.1002/alz.092734

**Published:** 2025-01-09

**Authors:** Rafael V. Lippert, Natalie C. Edwards, Giuseppe Tosto, Jeffrey D. Pyne, Patrick J. Lao, Adam M. Brickman

**Affiliations:** ^1^ Taub Institute for Research on Alzheimer’s Disease and the Aging Brain, Vagelos College of Physicians and Surgeons, Columbia University, New York, NY USA; ^2^ Columbia University Irving Medical Center, New York, NY USA; ^3^ Department of Neurology, Vagelos College of Physicians and Surgeons, Columbia University, and the New York Presbyterian Hospital, New York, NY USA; ^4^ Taub Institute for Research on Alzheimer’s Disease and the Aging Brain, Columbia University, New York, NY USA

## Abstract

**Background:**

Small vessel cerebrovascular disease (CVD), visualized as white matter hyperintensities (WMH) on magnetic resonance imaging (MRI), is associated with risk and progression of Alzheimer’s disease (AD) in clinical, community, and genetic studies of AD. However, it is unclear whether these observations indicate a role of CVD in AD pathogenesis. One approach towards understanding whether there is a mechanistic or fundamental function of CVD in AD pathogenesis is by examining whether genetic risk factors for AD are also associated with WMH. Here, we examined whether polygenic hazard scores (PHS) and cumulative incidence rates (CIR) scores are associated with WMH volume in older adults without dementia.

**Methods:**

We used data from the Alzheimer’s Disease Neuroimaging Initiative, including available WMH, PHS, and CIR scores from individuals without dementia. Polygenic hazard scores reflect the contribution of APOE and 31 other genetic variants to future risk for AD, calculated from over 54,000 AD cases and controls by Desikan and colleagues. The CIR scores combined the PHS and US population‐based incidence rates to reflect an age‐specific instantaneous risk for developing AD in each participant. Linear models were used to evaluate the relationship of PHS and CIR scores with log transformed WMH volumes. To confirm our findings among people with pathophysiological evidence of AD, we restricted the analysis to a subset of participants with amyloid positivity on amyloid PET scans.

**Results:**

Six hundred two participants were included (mean±SD age=72.6±7.12, 46% women). Polygenic hazard scores for AD were not associated with WMH volume (R=‐0.04, 95% CI:[‐0.12‐0.04], p=0.33), but higher CIR scores were associated with greater WMH volume (R=0.29, 95% CI:[0.21‐0.36], p<0.0001). The association between WMH and CIR remained after restricting the analyses to the amyloid positive subgroup (n=271, age=73.8±6.83, 51% women; R=0.20, 95% CI:[0.09‐0.31], p=0.0007).

**Conclusion:**

Higher cumulative incidence rate derived from polygenic risk scores for AD is related to WMH volume in individuals without dementia, suggesting that age‐anchored genetic risk for development of AD also increases small vessel CVD. The findings implicate WMH in the genetic pathway towards AD pathogenesis.

